# Effects of fasciatherapy versus fascial manipulation on pain, range of motion and function in patients with chronic neck pain

**DOI:** 10.1186/s12891-023-06769-0

**Published:** 2023-10-05

**Authors:** Syeda Aiman Batool, Syed Shakil-ul-Rehman, Zainab Tariq, Mehwish Ikram

**Affiliations:** Faculty of Rehabilitation and Allied Health Sciences, Riphah International University, Lahore, Pakistan

**Keywords:** Dannis bois method, Fascia therapy, Fascial manipulation, Chronic neck pain

## Abstract

**Background:**

Neck pain is among the common musculoskeletal problem that hinders a person’s daily activities. Fascial tightness is a familiar cause of chronic neck pain that is often neglected and can further cause neck disability and a limited range of motion.

**Objective:**

The purpose was to compare the effects of fascia therapy and fascial manipulation on pain, range of motion and function in patients with chronic neck pain.

**Methods:**

A randomized clinical trial was conducted from February to August 2022 in the Riphah Rehabilitation Centre, Lahore, Pakistan. Fifty-two participants of both genders, aged 18–40 years with chronic neck pain of at least 3–6 months were included. Group A (n = 26) received fascia therapy along with a conventional physical therapy protocol of hot pack, strengthening and stretching, while group B (n = 26) received the fascial manipulation treatment with conventional physical therapy. All the participants were assessed at baseline and after 3 weeks (3 sessions per week). Numeric Pain Rating Scale (NPRS), Neck Disability Index (NDI) and Goniometer (range of motions) were the outcome measures. SPSS 25 was used for the data analysis and normality of the data through the Shaphiro-Wilk test (p > 0.05), and parametric tests were applied.

**Results:**

The mean age of group A was 24.82 ± 2.64 years, and group B was 24.17 ± 2.20 years. The independent t-test result showed no significant difference (p ≥ 0.05) in all parameters except in cervical extension and right-side bending (p < 0.05). At the same time, the pair-wise comparison showed significant results (p < 0.05) for all outcome measures in both groups.

**Conclusion:**

DBM fascia therapy improved cervical extension and side bending (right) more than the fascial manipulation group.It is concluded that DBM fascia therapy shows more improvement as compared to other group.

**Trial registration number:**

This study was registered at ClinicalTrials.gov ID: NCT05272111 on 09/03/2022.

## Introduction

Neck pain is the fourth most common cause of neck disability and most people recover from it. Neck pain causes can be ruled out with a proper physical exam and history [1, 2]. Sometimes myofascial pain leads to muscle pain and myofascial trigger points and fascial restrictions are the causes of myofascial pain [3]. Many people experience neck pain at some point in their life [4]. Neck pain not only affects a person’s activity of daily living but also affects the quality of life [5]. Neck pain can be classified as acute if it is less than a period of 6 weeks, sub-acute (less than three months), and if the duration of neck pain is more than three to six months, then it is classified as chronic neck pain [2, 6]. There can be many risk factors and causes that can lead to neck pain which include poor posture [7], female gender, high job demands, and old age are some of them [6].

Many different treatment therapies are there for the improvement of painful symptoms around the neck [8], which include TENS [9], strengthening and stretching exercises [10] and mobilization of neck joints [11]. Among different causes of neck pain, fascia tightness is also one of the reasons that can also lead to painful neck ranges [12]. Fascia is a form of connective tissue which encloses muscles, tendons and nerves responsible for holding different organs together and has its own blood, lymph and nerve supply [13]. It is divided into four different layers based on its location. The superficial fascia is linked with skin; the deep fascia is connected to tendons and vessels; the visceral and parietal fascia is attached to internal organs [14]. Fascia tightness or irritation can cause pain, decreased range and reduced flexibility and can contribute to symptoms of shoulder head and neck pain [15]. Normal fascial mobility is necessary for normal musculoskeletal functioning [16].

The current study focuses on two different treatment techniques for improving chronic neck pain. The first technique is the DBM or Dannis Bois Method fascia therapy technique which French osteopath Prof. Dannis Bois developed. It is a non-manipulative soft tissue therapy technique that involves gentle pressure while stretching the body’s connective tissue. Three steps are involved in this technique: a somatic sense, gentle touch and body movement, which improves the elasticity of the tissues and decreases symptoms of a painful neck [17–19]. The second treatment technique in the present study that focuses on the deep fascia of the body is the fascial manipulation developed by Luigi Stecco PT. Stecco in the fascial model, divides the body into 14 segments in fascial manipulation: the head, neck, thorax, lumbar, pelvis, scapula, humerus, elbow, carpus, digits, hip, knee, foot and tarsus. Each segment was composed of six myofascial units (mf units) [20]. A painful point located in those myofascial units known as the centre of perception is identified and a special manipulative force is applied to that point usually located in the muscle belly to restore fascial elasticity and movement [21]. The heat generated from the manipulative force is used to restore movements of the elastin fibres of the fascia in the dense centre of fusion and coordination points [22]. The current study aims to compare the effects of fascia therapy and fascial manipulation on pain, range of motion (ROM), and function in patients with chronic neck pain. This study aims to offer evidence for fascia therapy and fascial manipulation that may enhance therapeutic techniques for persistent neck pain, particularly by emphasizing the fascial component. Also, it will provide evidence regarding these treatment techniques which might improve the treatment strategies for the management of chronic neck pain,

## Materials and methods

The randomized clinical trial (parallel group design) was conducted at Riphah Rehabilitation Centre, Lahore, Pakistan. After obtaining the ethical approval from the institutional ethics committee with a reference number of REC/RCR & AHS/22/0102. This study was registered at ClinicalTrials.gov ID: NCT05272111 on date 09/03/2022. The sample size of 52 was calculated after adding a 10% attrition rate using the epitool sample size calculator with a 5% variance and 95% confidence interval [23]. Using the convenience sampling technique (non-probability), the participants of both genders who followed the inclusion criteria of age 18–40 years with 3–6 months of neck pain (NPRS > 4)were randomly allocated into two groups by the lottery method. Each member was approached for the randomization method and then allocated to their respective groups.

Participants with a history of a recent surgery (3 months prior), neck trauma, systemic or soft tissue disease, pregnancy, radiculopathy and neck instability were excluded from the study. Both groups, A and B, received a three-week treatment session with three sessions per week on alternate days and the same baseline treatment therapy of 10 min hot pack, neck isometrics, and neck stretches in all planes.

### Group A

Group A received the DBM Fasciatherapy, a technique in which very gentle pressure was applied to a person’s body connective tissues. It is a non-manipulative technique in which soft and deep pressure is targeted to the connective tissues or fascia of the body. To apply this technique the patient was comfortably sitting on a seat then through the application of the specialized touch of the therapist, involving the patient’s somatic sense, and the specific body movement of the patient were the three steps that were involved in the fascia therapy. Along with this, the conventional treatment involves the hot pack (10 min), neck flexion, extension, side bending and rotation isometrics (each with 10-sec hold, 5–6 reps), with stretching of the neck flexors, extensors, side benders and rotators (each with a 10-sec hold, 5–6 reps) were given. A total of 45 min sessions three times a week on alternate days for three weeks were given to these patients [3, 19, 24].

### Group B

Group B received the fascial manipulation technique that involves the application of the appropriate manipulation on the specific point of the fascia with limited movement. The patient was comfortably lying on the plinth with the therapist on the head side. The therapist locate the specific points on the fascia anteriorly, posteriorly, and posterolaterally. Then a specific oscillatory manipulative force was applied directly over that point to resolve that fascia tightness. Along with this, the conventional treatment involves the hot pack (10 min), neck flexion, extension, side bending, and rotation isometrics (each with a 10-sec hold, 5–6 reps) with neck flexors, extensors, side benders and rotators stretching (each with a 10 s hold, 5–6 reps) were given. A total of 45 min sessions three times a week on alternate days for three weeks was given to these patients [3, 20, 24].

All the participants in both groups were assessed for pain using the NPRS scale. NPRS values ranged from 0 to 10, zero shows no pain and 10 shows worst pain and reliability was ICC = 0.85–0.96 [25]. Functional disability was assessed by the NDI Urdu version. NDI is a 10-item questionnaire used to measure the functional disability of the neck. A higher NDI-U score indicated that the patient is having more disability (maximum score 50) and has excellent reliability ICC > 0.90 [26, 27]. The range of motion of cervical flexion, extension, left and right side bending, and left and right-side rotation of the neck was measured by a goniometer. The reliability of the goniometer ranges from 0.92 to 0.99 [28]. The outcome assessor and participants were blinded from the allocation of the treatment protocol. It was hypothesized that there was a difference between the effects of fasciatherapy versus facial manipulation on pain, range of motion and function in patients with chronic neck pain.

Descriptive and statistical tests were applied using SPSS, IBM version 25. For the quantitative variables, mean and standard deviation were calculated. Results of the Shapiro-Wilk test (p > 0.05) showed that the data were normally distributed, so parametric tests were applied to evaluate the effects of fasciatherapy and fascial manipulation on patients with chronic neck pain. The paired t-test shows differences within the group while the differences across the group were shown by the independent t-test and for significant differences, the p-value was set as p ≤ 0.05.

## Results

**Fig. 1:**
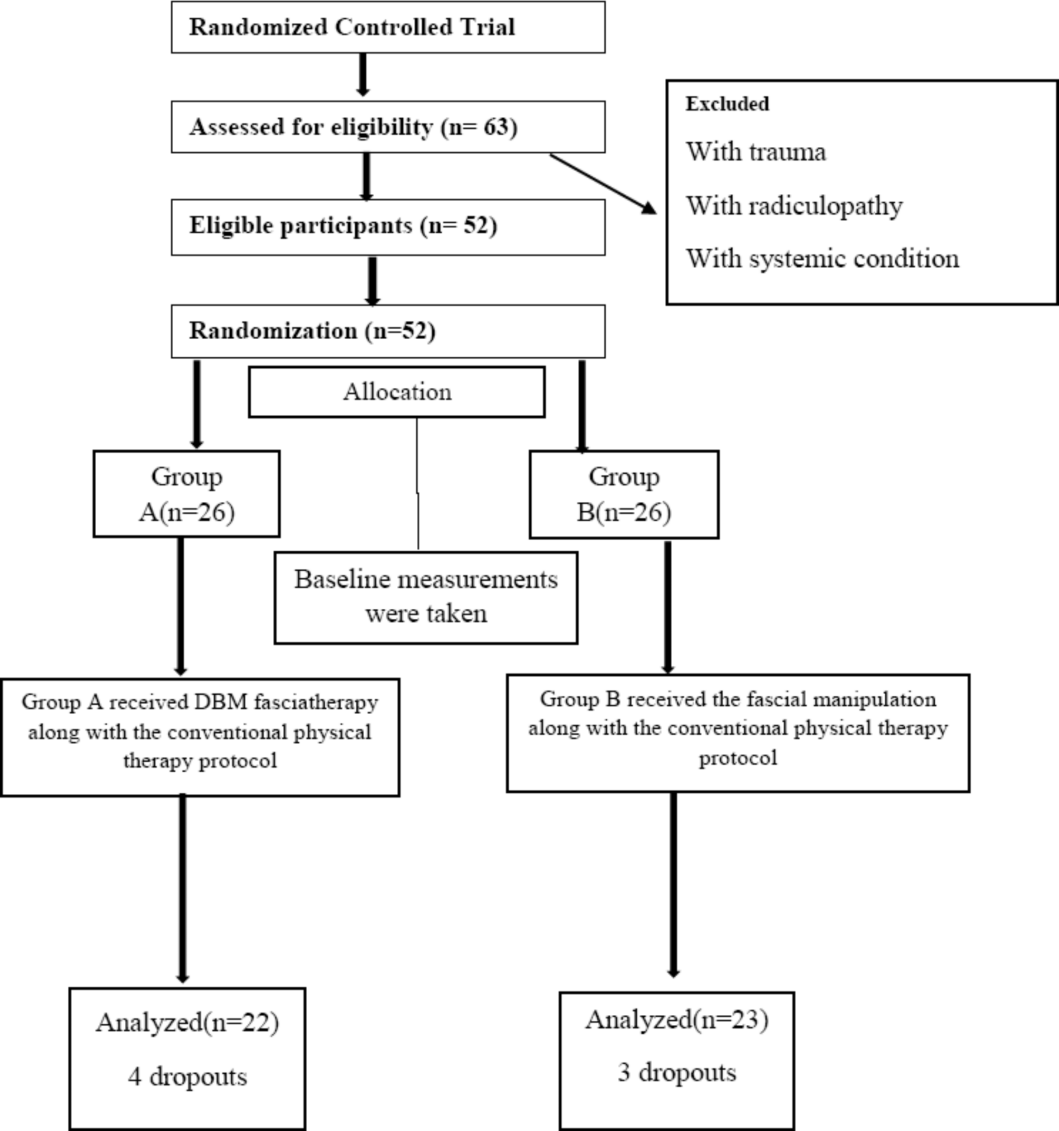
Flow Chart

After screening 63 patients, 52 participants who fulfilled the inclusion criteria were included in the study. Participants were randomly allocated into two groups using the lottery method. There were 26 participants in group A and 26 in group B, with a mean age of 24.82 ± 2.46 and 24.17 ± 2.20 in groups A and B, respectively. All the participants received treatment for three weeks with three sessions per week on alternate days. The data was analysed using SPSS version 25. There were four dropouts from Group A and three from Group B. The CONSORT flow diagram is shown in Fig. 1.

Demographic characteristics are shown in Table 1. Table 2 shows the Across and Within-group analysis for pre and post-treatment values of both groups by independent and paired sample t-test for the variable of pain (NPRS), function (NDI) and cervical flexion and extension. Cervical extension showed a significant result (p < 0.05) that indicates group A was more effective in improving cervical extension while in other parameters (pain, function and cervical flexion) there was no significant difference (p > 0.05). At the same time, the Within-group analysis showed that both treatments were effective in improving pain, function and cervical ranges.

**Table 1:** Baseline Demographics of Both Groups

Baseline characters	DBM group (A)	Fascial manipulation group (B)
**No. of participants**	22	23
**Gender**	Males = 1 Females = 21	Males = 1 Females = 22
**Mean age**	24.82 ± 2.48	24.17 ± 2.20

**Table 2:** Across and within-group comparison of NPRS, NDI, Cervical Flexion and Extension

	Group A (Mean ± S.D)	Group B (Mean ± S.D)	Mean Difference	P-value
**Pre-NPRS**	6.09 ± 0.61	6.04 ± 0.63	0.05	0.98
**Post-NPRS**	2.54 ± 0.67	2.89 ± 0.75	-0.35	0.92
**Mean Difference**	3.55	3.15		
**P-value**	0.00	0.00		
	**Group A** **(Mean ± S.D)**	**Group B** **(Mean ± S.D)**	**Mean Difference**	**P-value**
**Pre-NDI**	48.45 ± 3.90	49.64 ± 3.01	-1.19	0.38
**Post-NDI**	22.54 ± 3.01	23.13 ± 3.50	-0.59	0.26
**Mean Difference**	25.91	23.51		
**P-value**	0.00	0.00		
	**Group A** **(Mean ± S.D)**	**Group B** **(Mean ± S.D)**	**Mean Difference**	**P-value**
**Pre-Cervical Flexion**	68.63 ± 5.60	71.52 ± 4.37	-2.89	0.32
**Post-Cervical Flexion**	82.27 ± 4.81	85.0 ± 4.26	-2.73	0.09
**Mean Difference**	-13.64	-13.48		
**P-value**	0.01	0.00		
	**Group A** **(Mean ± S.D)**	**Group B** **(Mean ± S.D)**	**Mean Difference**	**P-value**
**Pre-Cervical Extension**	49.77 ± 8.92	54.56 ± 5.41	-4.79	0.5
**Post-Cervical Extension**	66.36 ± 3.51	66.52 ± 3.82	-0.22	0.02
**Mean Difference**	-16.59	-11.96		
**P-value**	0.00	0.00		

Abbreviations:NPRS = Numeric Pain Rating Scale; NDI = Neck Disability Index

Table 3 shows the Across and Within-group analysis for pre and post-treatment of both groups by independent and paired sample t-test for the variable of cervical side-bending and rotation (right and left). There was no significant difference (p > 0.05) in cervical rotation (right and left) and side bending (left) but cervical side-bending (right) showed a significant result (p < 0.05) that indicates group A was more effective in improving cervical side-bending. While the Within-group analysis showed that both treatments significantly improved (p < 0.05) cervical side-bending and rotation (both left and right).

**Table 3:** Across and Within-group comparison of cervical side-bending and rotation

	Group A (Mean ± S.D)	Group B (Mean ± S.D)	Mean Difference	P-value
**Pre-Side Bending (Right)**	30.45 ± 6.88	24.34 ± 4.07	6.11	0.83
**Post-Side Bending (Right)**	41.36 ± 3.83	38.69 ± 4.32	2.67	0.02
**Mean Difference**	-10.91	-14.35		
**P-value**	0.00	0.01		
	**Group A** **(Mean ± S.D)**	**Group B** **(Mean ± S.D)**	**Mean Difference**	**P-value**
**Pre-Side Bending (Left)**	30 ± 5.11	26.52 ± 2.79	3.48	0.45
**Post-Side Bending (Left)**	41.36 ± 3.51	39.78 ± 3.52	1.58	0.12
**Mean Difference**	-11.36	-13.26		
**P-value**	0.00	0.00		
	**Group A** **(Mean ± S.D)**	**Group B** **(Mean ± S.D)**	**Mean Difference**	**P-value**
**Pre-Rotation (Right)**	70.45 ± 5.32	70.86 ± 4.96	-0.41	0.64
**Post-Rotation (Right)**	82.04 ± 2.95	83.69 ± 4.57	-1.65	0.17
**Mean Difference**	-11.59	-12.83		
**P-value**	0.04	0.00		
	**Group A** **(Mean ± S.D)**	**Group B** **(Mean ± S.D)**	**Mean Difference**	**P-value**
**Pre-Rotation (Left)**	69.31 ± 3.87	71.30 ± 3.75	-1.99	0.49
**Post-Rotation (Left)**	82.27 ± 3.35	80.34 ± 2.74	1.93	0.35
**Mean Difference**	-12.96	-9.04		
**P-value**	0.00	0.04		

## Discussion

The current study aimed to compare the effects of two treatment techniques fascia therapy and fascial manipulation. Fascia therapy improved the cervical ranges; extension and right side bending, but there was no difference in other parameters. Fascia therapy and fascial manipulation techniques were equally effective in improving pain, range of motion and function in neck pain management. Both groups were assessed for NPRS, NDI, and cervical ranges. Cervical extension and right-side bending showed significant results (p ≤ 0.05), which means group A (DBM fascia therapy) was more effective, but in the case of all other parameters, there were non-significant results (p ≥ 0.05).

In a previous study the fascial manipulation (FM) technique was used on thirty students with neck pain. Both groups were assessed for NPRS, NDI, and cervical ranges. Similar results are shown in a study that fascial manipulation and combination therapy (ultrasound and tens) can be used for neck pain management as both show significant improvement [26]. Another study shows similar effects as the present study that there was a significant reduction in pain intensity and improvement in the VAS score when fibromyalgia patients were treated for 5–15 sessions of fascia therapy [29]. In a study where chronic low back patients were treated with fascial manipulation (FM). There was a significant difference (p < 0.05) in the patient’s pain level, functional level, and flexibility before and after the treatment. FM provided significant pain relief after the treatment and these findings are consistent with this study where there is a significant improvement in pain and range after the FM treatment [30].

There was a reduction in pain levels and psychological suffering in the patients to whom DBM fascia therapy was given as a part of the treatment [17], the results are consistent with the current study but different in respect that the psychological aspect was not monitored. Another study’s findings are in line with the current study that after the treatment application, fascial manipulation provided significant pain relief for chronic low back patients [31]. A systematic review to investigate the efficacy of fascial manipulation techniques in patients with different musculoskeletal conditions was conducted. Fascial manipulation had moderate effects in improving pain and disability among patients with musculoskeletal conditions [32].

The perception of the clinical utility and awareness of the benefits of using DBM fascia therapy in the management of pain was explored. The self-structured questionnaire was sent to French physiotherapists who were using DBM fascia therapy as a treatment regime for patients with pain. DBM Fasciatherapy-trained physiotherapists who practised it in their clinical settings showed improvement in the symptoms of both physical pain and psychical suffering. This treatment technique resulted significantly in headaches, neck and lower back pain, and migraines [19], the current study results also show improvement in pain after fasciatherapy. The effects of DBM fascia therapy were seen on the fascial systems of the thoracolumbar fascia, crural fascia, thoracolumbar, and pectoralis major aponeurosis with the help of elastography. Three groups receive high-speed manipulation, low-speed manipulation, and low-speed manipulation on supporting points; the elastography shows that the individuals receiving the DBM fascia therapy have improved fascial layering and blood flow [18]. Myofascial Release Therapy (MFR) was an effective treatment technique in improving ROM and reducing the symptoms in patients with mechanical neck pain as compared to the conventional physical therapy protocol [33]. The effects of Dannis Bois’s method of fascia therapy, reflexology and hypnosis, and music therapy were seen in patients dealing with daily life stress and anxiety. Fascia therapy, reflexology, and hypnosis therapy significantly reduced anxiety and stress levels and could be used as a non-pharmacological treatment intervention protocol [34].

Fascia therapy and fascial manipulation both positively affect tissues and muscles and relax the body physically and psychologically. The neck region is especially affected when the patient is in stress emotionally. Trigger points and tight muscles lead to severe neck pain, but the myofascial release will benefit both acute and chronic cases.

The limitation of this study was the participation of more females in this study and only two males participated. Another limitation was that the duration of treatment was of 3 weeks as in many studies, it ranged from 6 to 8 weeks for better results but in a few studies, 2 weeks was also a suitable period for myofascial release. In future, we can add a long duration of treatment sessions with follow-ups.

## Conclusion

According to the study findings, DBM fasciatherapy shows more improvement in cervical extension and right-side bending range of motion as compared to the fascial manipulation technique. Although there was no statistically significant difference between the groups in other parameters. Both types of treatment techniques showed clinical improvement in neck pain intensity, disability and range of motion.

## Data Availability

Data will be available at a reasonable request from the corresponding author.

## Abbreviations

DBM: Dannis Bois Method
MFR: Myofascial Release
FM: Fascial Manipulation
NPRS: Numeric Pain Rating Scale
NDI: Neck Disability Index
CONSORT: Consolidated Standards of Reporting Trials

## References

[1] Safiri S, Kolahi A-A, Hoy D, Buchbinder R, Mansournia MA, Bettampadi D et al. Global, regional, and national burden of neck pain in the general population, 1990–2017: systematic analysis of the global burden of disease study 2017. BMJ. 2020;368. 10.1136/bmj.m791 PMC7249252 32217608

[2] Popescu A, Lee H (2020). Neck pain and lower back pain. Med Clin.

[3] Pawlukiewicz M, Kochan M, Niewiadomy P, Szuścik-Niewiadomy K, Taradaj J, Król P, Kuszewski MT (2022). Fascial manipulation method is effective in the treatment of Myofascial Pain, but the treatment protocol matters: a Randomised Control Trial—Preliminary Report. J Clin Med.

[4] ul Abideen MZ, Afzal W, Tanveer F, Rafique O, Ahmad A, Malik A (2018). Prevalence of neck pain in goldsmiths. Rawal Med J.

[5] Monticone M, Ambrosini E, Rocca B, Cazzaniga D, Liquori V, Pedrocchi A (2017). RETRACTED: Group-based multimodal exercises integrated with cognitive-behavioural therapy improve disability, pain and quality of life of subjects with chronic neck pain: a randomized controlled trial with one-year follow-up. Clin Rehabil.

[6] Kim R, Wiest C, Clark K, Cook C, Horn M (2018). Identifying risk factors for first-episode neck pain: a systematic review. Musculoskelet Sci Pract.

[7] Bakry HA (2021). The effect of poor posture on the cervical range of motion in young subjects. Egypt J Phys Ther.

[8] Sebastian D. Principles of manual therapy. Jaypee Brothers Medical Publishers; 2019.

[9] Díaz-Pulido B, Pérez-Martín Y, Pecos-Martín D, Rodríguez-Costa I, Pérez-Muñoz M, Calvo-Fuente V (2021). Efficacy of manual therapy and transcutaneous electrical nerve stimulation in cervical mobility and endurance in subacute and chronic neck pain: a randomized clinical trial. J Clin Med.

[10] Parikh P, Santaguida P, Macdermid J, Gross A, Eshtiaghi A (2019). Comparison of CPG’s for the diagnosis, prognosis and management of non-specific neck pain: a systematic review. BMC Musculoskel Disord.

[11] Coulter ID, Crawford C, Vernon H, Hurwitz EL, Khorsan R, Booth MS (2019). Manipulation and mobilization for treating chronic nonspecific neck pain: a systematic review and meta-analysis for an appropriateness panel. Pain Physician.

[12] Hrkal P, Fascia (2015). The Tensional Network of the human body: the science and clinical applications in manual and movement therapy. J Can Chiropr Assoc.

[13] Bordoni B, Mahabadi N, Varacallo M, Anatomy. fascia. 2018. 29630284

[14] Gatt A, Agarwal S, Zito PM (2021). Anatomy, fascia layers.

[15] Prabu Raja G, Shyamasunder Bhat N, Marie Cruz A, Prabhu A, Fernandes S, Naaz N (2022). The anatomical myofascial continuum between the neck and eyes. Clin Anat.

[16] Langevin HM (2021). Fascia mobility, proprioception, and myofascial pain. Life.

[17] Courraud C, Bertrand I, Dupuis C, Bois DC. DBM Fasciatherapy and Pain: the Practitioners’Perspective.

[18] Courraud C, Bertrand I, Dupuis C. Assessment of the effects of DBM fasciatherapy on fascial system with elastography.

[19] Courraud C, Lieutaud A, Bertrand I, Dupuis C, Bois D (2021). Practitioner utilization and perceptions of the clinical utility of Danis Bois Method (DBM) Fasciatherapy to pain management: a survey of french physiotherapists. Adv Integr Med.

[20] Podstawka Z, Pińkowska O, Byś A, Gawda P (2020). Effectiveness of Fascial Manipulation Method (FM®). J Educ Health Sport.

[21] Mathew NP, Davis F. Effect of Fascial Manipulation on Glenohumeral Internal Rotation Deficit in overhead Athletes-A Randomized Controlled Trial. Muscles, Ligaments Tendons J (MLTJ). 2020;10(1).

[22] Raja GP, Bhat NS, Fernández-de-las-Peñas C, Gangavelli R, Davis F, Shankar R (2021). Effectiveness of deep cervical fascial manipulation and yoga postures on pain, function, and oculomotor control in patients with mechanical neck pain: study protocol of a pragmatic, parallel-group, randomized, controlled trial. Trials.

[23] Rodríguez-Huguet M, Rodríguez-Almagro D, Rodríguez-Huguet P, Martín-Valero R, Lomas-Vega R (2020). Treatment of neck pain with myofascial therapies: a single blind randomized controlled trial. J Manipulative Physiol Ther.

[24] Rodríguez-Huguet M, Rodríguez-Almagro D, Rodríguez-Huguet P, Martín-Valero R, Lomas-Vega R (2020). Treatment of neck pain with myofascial therapies: a single blind randomized controlled trial. J Manip Physiol Ther.

[25] Siddiqui M, Akhter S, Baig AAM (2022). Effects of autogenic and reciprocal inhibition techniques with conventional therapy in mechanical neck pain–a randomized control trial. BMC Musculoskelet Disord.

[26] Farooq MN, Mohseni-Bandpei MA, Gilani SA, Hafeez A (2017). Urdu version of the neck disability index: a reliability and validity study. BMC Musculoskelet Disord.

[27] Vernon H, Mior S. The Neck Disability Index: a study of reliability and validity. J Manipulative Physiol Ther. 1991. 1834753

[28] Nastiti AZ, THE EFFECTIVENESS OF FASCIAL MANIPULATION FOR NECK, PAIN AMONG UNIVERSITY STUDENTS (2020). J Asian Orthop Manipulative Phys Ther.

[29] Dupuis C. Fibromyalgia, pain and DBM Fasciatherapy.

[30] Endamlı D, Bayramlar K, Turhan B (2019). Investigation of fascial treatment effectiveness on pain, flexibility, functional level, and kinesiophobia in patients with chronic low back pain. Physiother Q.

[31] Doğan BE, Bayramlar K, Turhan B (2019). Investigation of fascial treatment effectiveness on pain, flexibility, functional level, and kinesiophobia in patients with chronic low back pain. Physiother Q.

[32] Arumugam K, Harikesavan K (2021). Effectiveness of fascial manipulation on pain and disability in musculoskeletal conditions. A systematic review. J Bodyw Mov Ther.

[33] Gauns SV, Gurudut PV (2018). A randomized controlled trial to study the effect of gross myofascial release on mechanical neck pain referred to upper limb. Intern J Health Sci.

[34] Payrau B, Quere N, Breton E, Payrau C (2017). Fasciatherapy and reflexology compared to hypnosis and music therapy in daily stress management. Int J Ther Massage Bodyw.

